# Supplementary figures and images for: Circadian patterns of heart rate, respiratory rate and skin temperature in hospitalized COVID-19 patients
Source: PLoS One. 2022 Jul 7;17(7):e0268065. doi: 10.1371/journal.pone.0268065 (PMC9262173; doi:10.1371/journal.pone.0268065)

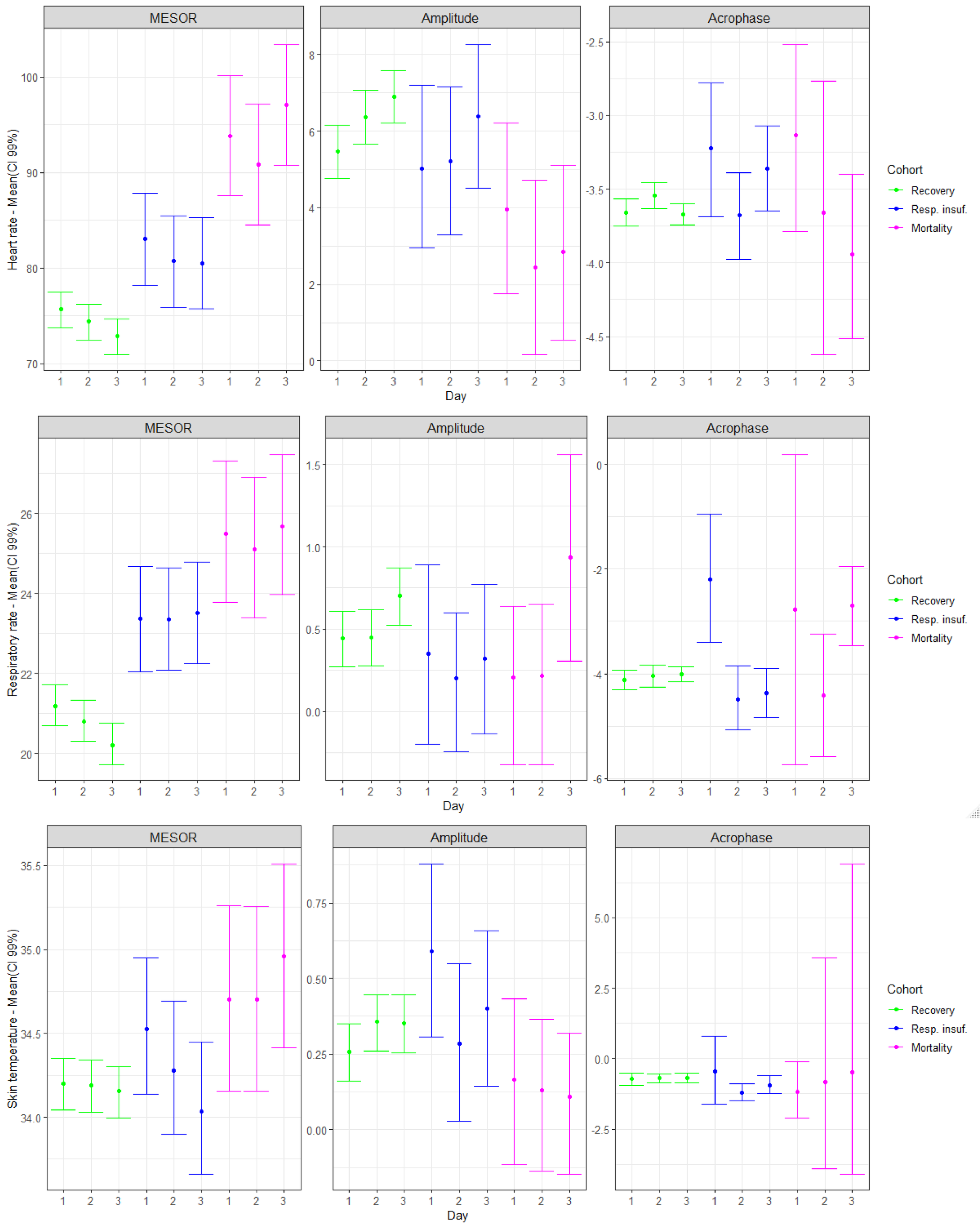

Supplement: S1 Fig — MESOR: midline estimation statistic of rhythm. MESOR and amplitude are in /min for heart rate and respiratory rate, and°C for skin temperature. Acrophase in (degree). (TIF) [file pone.0268065.s002.tif]
