# Supplementary material for: Circadian patterns of heart rate, respiratory rate and skin temperature in hospitalized COVID-19 patients
Source: PLoS One. 2022 Jul 7;17(7):e0268065. doi: 10.1371/journal.pone.0268065 (PMC9262173; doi:10.1371/journal.pone.0268065)
Supplement: Attachment — Submitted filename: PONE-D-21-34906_R1.pdf [file pone.0268065.s004.pdf]

Review for PONE-D–21–34906

In this retrospective study among 429 covid–19 patients admitted to the general ward, the authors aim to evaluate whether circadian rhythms can be observed in those patients with respect to heart rate, respiratory rate and skin temperature. Moreover, the authors explore a possible association between circadian pattern amplitude and hypoxic respiratory insufficiency. Data from five predefined time intervals were analyzed: (1) the first and (2) last three days of admission, the three days (3) preceding and (4) succeeding hypoxic respiratory insufficiency, and the (5) last three days before death.
Results revealed that heart rate and respiratory rate followed a circadian pattern in all stages of hospital admission, except for the days prior to death. Skin temperature only followed a circadian pattern on admission, discharge, and the days preceding respiratory insufficiency.
The authors conclude that these circadian patterns could improve monitoring- and alarm strategies. However, the predictive power of peak-nadir excursion however appears to be low.

The authors are to be complimented for their initiative to provide data in this important and new field of research. However, there are significant issues regarding explanation of methods, data analysis and the presentation of the results. Furthermore, part of the conclusion does not seem to be supported by the data provided.

Major Comments
1. We ask the authors to explain in more detail the rational for the definition of each predefined stages/cohorts? Why did the authors did not perform a longitudinal statistical analysis? It would be interesting to know if circadian patterns change over time depending on specific covariates (covariate analysis: e.g. respiratory insufficiency as a covariate). We suggest a separate statistical methods paragraph. Why are p-values not provided within the manuscript. A review by an independent statistician may be helpful.
2. The applied definition of *a circadian pattern* should be explained in the manuscript. Sentences like *A circadian pattern was observed in HR and RR during stages I-IV.* are unclear. When should clinicians deem a pattern as a *normal circadian rhythm*? Did the authors look for indicators of circadian disturbances when analysing the data? A longitudinal analysis of patterns comparing patients with and without developing respiratory insufficiency would be very interesting for the readership.
3. The results section is very hard to “digest”; especially for readers who are not experts in the s very new field of research. We strongly suggest to amend the results section with figures, which illustrate the main results more clearly.
4. The authors conclude that circadian patterns of heart rate, respiratory rate and skin temperature could improve monitoring- and alarm strategies. At this stage of the manuscript, we do not think that this conclusion is supported by the data. We agree, that including informations of circadian profiles within in prediction models and monitoring algorithms may be an interesting and helpful approach in the future. We ask the authors to provide 1 or 2 concrete examples or clinical scenarios how this could be helpful in clinical decision making. However, the authors should be clear, that this is not supported by their data and should remove statements from the conclusion section.
5. Page 10, Line 204: „The mean circadian pattern amplitude showed differences between stages, but only an increasing PNex of skin temperature was associated with developing respiratory insufficiency and with only a small effect size." Please provide a P-value. Was the association statistical significant?

Further Comments
- Page 3, Line 42–44: „Since September 2020, patients with covid–19 are treated with dexamethasone, which has a suppressive effect on the circadian pattern of the human metabolism." What does *suppressive effect on circadian pattern* mean? Please explain.
- Page, Line 226: „The decrease of circadian rhythm might be due to severe illness and extreme physical stress, but could also have been influenced by age, comorbidities and medication. Furthermore, circadian rhythms are highly influenced by light input. As part of palliative care, patients were often relocated to single rooms with closed blinds for comfort. This might have played a role in the decrease of circadian pattern seen in all vital parameters.“ Please explain in more detail why circadian rhythm disturbances can occur in the context of severe diseases, e. g. closed eyes, reduced physical activity, inflammation etc.
