# Supplementary material for: Circadian patterns of heart rate, respiratory rate and skin temperature in hospitalized COVID-19 patients
Source: PLoS One. 2022 Jul 7;17(7):e0268065. doi: 10.1371/journal.pone.0268065 (PMC9262173; doi:10.1371/journal.pone.0268065)
Supplement: Attachment — Submitted filename: PLOS ONE D21-34906 notes.docx [file pone.0268065.s003.docx]

PLOS ONE – D 21 34906

**Circadian patterns of HR, RR, and skin temp in hospitalized COVID19 patients**

This study evaluated circadian patterns in patients with COVID19 admitted to inpatient ward, using pulseox + “wireless sensor” measurements over 5 different time windows of their hospitalized illness. As a descriptive study this is interesting and provides rationale for continuing to investigate the utility of such measurements for risk prediction and patient monitoring.

The main issue is that the current study design does not support an analysis of *risk* for hypoxemic respiratory insufficiency as the authors propose. Evaluating different time periods a single cohort of patients, comparing a pre-hypoxemic “control” period and post-hypoxemic “outcome” period in those who developed hypoxemic respiratory insufficiency (defined in this study as >15L O2 therapy), we are able to establish only that these measurements differ in these two periods of time within these patients. To evaluate for *risk* of hypoxemic respiratory insufficiency, the authors must include a study population that did not develop hypoxemic respiratory insufficiency. This could be done in a variety of ways to create either a cohort or a case-control study design. The analysis should be revised to include such a cohort, or the manuscript needs to be rewritten with this significant change in mind.

It would also be helpful to include references using the methods employed (daytime peak vs. nighttime nadir of physiologic data) to evaluate circadian patterns in the data.

Abstract

- Please define “delta day” as a measurement in the abstract.

- Please revise to more accurately describe the logistic regression results (that VS changes are associated with worsened hypoxemia within in this select cohort of patients; they do not demonstrate risk of developing hypoxemia – as above, this cannot be evaluated unless the study also includes patients who did not develop hypoxemic respiratory insufficiency.)

Methods

- p5. Please clarify - if sT measurements were not directly available then how were they studied?

- Please include how many COVID19 patients were excluded for lack of sensor data.

- p.6. Please correct “improbably data.”

- The authors excluded patients with < 48 hours of continuous data during a three-day period, except for the post-hypoxemic respiratory failure period. This suggests that patients who developed hypoxemic respiratory failure very quickly (eg after 1-2 days) or who died shortly after admission without developing hypoxemic respiratory failure, etc. would have been excluded. This biases the study population to those patients who had a relatively slow progression of disease and may bias the study population away from those admitted to the ICU (it is unclear whether remote monitoring continued after ICU admission?). The authors should consider revising the analysis, using a shorter time window for each “period”.

- Consider including a flow diagram to describe the total number of potentially eligible patients, and those excluded at each stage (e.g. as suggested by the STROBE statement).

- Including patient data in more than one time-period “cohort” and then comparing differences between time periods is problematic from a study design perspective. This introduces bias towards the null – which makes it more difficult to identify true differences in vital signs between cohorts. The authors should consider revising their compared time periods so that no overlap occurs.

- How accurately was the onset of hypoxemic respiratory insufficiency defined? It would be helpful to describe how this information was obtained, e.g. if this was time stamped (hour and minute) to ensure that the vital sign data was assigned correctly as pre- vs. post-intervention.

- p.7. Were peaks and nadirs drawn from raw data? Typically, circadian analyses using peak/nadir are drawn from a cosinor or sinusoidal analysis that accounts for the entire dataset shifting + and – in a circadian fashion, which is less sensitive to random noise (e.g., Shoben Am J Epi 2011 for an excellent example.) If no cosinor analysis is applied, thenit seems like this peak-nadir measurement (PNex) will only provide the range of daytime vs. nighttime data. Please include references for using this measurement in circadian analyses.

Results:

- p. 7. Please rename the 5 time periods; they are not truly “cohorts” as they include the same patients, and the patient data is actually duplicated across some of these analytic groups. Suggest using “time periods” or something similar.

- How do more patients have respiratory insufficiency than deterioration? This might be clarified by using more distinct/separated definitions of time periods for comparison and/or a flow diagram as suggested above.

- Table 3 sugests that day 1 vs. 2 of respiratory insufficiency and day 1 vs 3 of respiratory insufficiency were compared in logisitc regression. This is different from the analyses described in the Methods. Please clarify.

-p.9; was mean PNex compared quantitatively between stages using a statistical test? If so, consider adding these comparisons to table 2.

-p.9, lines 189-191 and 191-192 should be edited for accuracy. Since patients without hypoxemic respiratory insufficiency were not included in this analysis, we cannot evaluate whether this development was associated with the development of hypoxemia. All we know is that the delta PNex was associated with skin temperature in this select cohort of patients who will go on to develop respiratory insufficiency.

-Table 3: Table 3 compares the difference in Pnex between days 1 and 2 and days 1 and 3. Please clarify – is this limited to stage III patients? If so, please add this detail and the number of patients included to the title.

Discussion:

- I agree that Figure 1 suggests qualitatively that there is circadian variability given the cyclical nature of overall VS pattern. However, it’s hard to claim definitively that there is no circadian variability based on a subjective assessment (eg for the patients with mortality.) From figure 1, stage I and stage III do not necessarily look all that different – it is not clear what the authors used as a cut off for circadian rhythm presence vs. absence. If a quantitative analysis is possible, one should be included. If not, the discussion should reflect this uncertainty throughout.

- The Discussion should be edited in several places to accurately reflect the results presented in Table 3. This analysis supports only an association between PNex and skin temperature, not an association with hypoxemic respiratory failure.

- If the PNex is not a validated method of measuring circadian amplitude, then the related portions of the discussion should be revised accordingly.

Figure 1

- Please provide a legend for the green, red, and black tracings. The caption says that the Y axis is hours; is this correct? (It seems like the X axis is hours?)

Figure 2

- Consider defining in the methods that you plan to compare day 1, 2 and 3 within the 5 time windows. Were any quantitative analyses applied to these 1/2/3 day comparisons? Clarifying why it was included and what these results mean would be helpful.
