# Supplementary material for: Circadian patterns of heart rate, respiratory rate and skin temperature in hospitalized COVID-19 patients
Source: PLoS One. 2022 Jul 7;17(7):e0268065. doi: 10.1371/journal.pone.0268065 (PMC9262173; doi:10.1371/journal.pone.0268065)
Supplement: Attachment — Submitted filename: Response to comments - Circadian patterns in covid-19.docx [file pone.0268065.s005.docx]

**Circadian patterns of heart rate, respiratory rate and skin temperature in hospitalized covid-19 patients -** Response to reviewers’ and editors’ comments

**EDITOR**

**Fix issues with citations.**

We have critically revised our references, and have adjusted citations in accordance with the suggestions made by the reviewers.

**Clarify information on dexamethasone treatment and absent data.**

We have added information on dexamethasone treatment, and have provided information on the amount of continuous data used in analysis.

**Provide more details on methodology and statistics as specified by the reviewers.**

We have considerably modified the analysis methodology as per the reviewers’ suggestions, and have added more elaborate explanations and references to substantiate our choices.

**Improve discussion about alternative methodologies and include more recent references.**

We have added references to the new methodological approach, which was recently been developed and has been used to assess circadian rhythm in HRV in covid-19 patients. We have added several more recent references in the discussion, especially regarding covid-19.

**Consider including a study population that did not develop hypoxemic respiratory insufficiency – or at least discuss this omission and tone down conclusions.**

We have redefined our cohorts to clarify the distinction between patients that did and did not experience respiratory insufficiency.

**Consider including a flow diagram to describe the total number of potentially eligible patients, and those excluded at each stage (e.g. as suggested by the STROBE statement).**

We have added a flow chart (figure 1) of patient inclusion.

**Avoid including patient data in more than one time-period “cohort” and then comparing differences between time**

We have redefined our cohorts to clarify the distinction between patients that did and did not experience respiratory insufficiency, and have aimed to model coefficient changes over time.

**If no cosinor analysis is applied, thenit seems like this peak-nadir measurement (PNex) will only provide the range of daytime vs. nighttime data. Please include references for using this measurement in circadian analyses.**

We have no longer used the method of PNex, but used cosinor modelling as suggested by several reviewers.

**Include a quantitative analysis for rhythmicity.**

We have added a rhythmicity test based on the goodness of fit of the cosinor model for the population mean cosinor models.

**Discuss more specifically how findings could be translated into the clinics.**

We have specifically addressed the implications for both predictive modelling and clinical practice.

**Reviewer #1

1. Introduction: “Since September 2020, patients with covid-19 are treated with dexamethasone[15], which has an suppressive effect on the circadian pattern of the human metabolism[16].” The citation (16) is not applicable. In the cited study dexamethasone was administered in the afternoon. Within the clinical routine, dexamethasone is likely to be administered in the early morning which might rather result in a strengthening of circadian rhythms. How many patients received dexamethasone in this study? Why September 2020, not June 2020?**

Thank you very much for this observation. We did not realize the difference in timing of administration of dexamethasone would have the opposite impact in our study population. We have adjusted the sentence to better reflect this. As for the amount of patients receiving dexamethasone, we have added this to our baseline table. We chose September 2020 since this was the date of the first scientific publication of the RECOVERY trial according to the website of the RECOVERY trial. We have adjusted this to July 2020 when the first preliminary report was published by the NEJM.

**2. Methods: “Inclusion of patients stopped because the wearable sensor was no longer available.” Please specify: Was the availability of the sensor tied to funding for the study or did the company stop production due to unreliable measurement accuracy?**

Unfortunately, the reason behind the availability of the sensor is too long a story to add to this paper. The wearable we used is reliable (Selvaraj et al., 2018). The distributor of the wearable (Philips) however had developed their own patch sensor and stopped the production of the old sensor (the one we used). Unfortunately, that new sensor turned out to be not reliable. The distributor then decided to stop selling these sensors all together and instead focused on a new type of sensor, leaving us without sensors. We have added a summary of the reason to the paper. Hopefully this provides enough clarification.

**3. Methods: “Patients with a pacemaker did not receive a sensor since RR measurements might result unreliable in paced rhythms.” Please clarify: Were those patients completely excluded from the study or only RR measurements were excluded for those patients?**

Since patients with a pacemaker did not receive a sensor, they had no available continuous data and therefore did not meet the inclusion criteria as described in the paragraph on ‘data collection’. We have added ‘sensor’ to the notion of ‘available continuous data’ to clarify that only patients who received a sensor could be included.

**4. Methods: „Since our cohort included dying patients, we used wide limits for improbably 114 data (for RR <1/min & >80/min; for HR <30/min & >280/min; for sT < 25°C).” The lower limits for temperature and respiratory rate are extremely wide and should be critically revised.**

Thank you for this feedback, we have discussed this extensively. The limits were chosen based on literature and clinical experience, but also on observations in the data. Especially in patients who were monitored while dying, it is hard to say which values are still valid and which are not. Therefore we have chosen to use wide limits, but also use additional methods to filter out possible artefacts (by deleting short lasting changes in HR and RR, and the ‘warming up’ period of skin temperature), and using a median filter of 15 minutes, before performing further analyses.

**5. Methods: “Data was divided in five cohorts based on different stages of disease…. Hypoxic respiratory insufficiency was defined as the need for 15 l/min oxygen therapy.” Why did the authors not stratify according to the WHO criteria in mild, intermediate and severe COVID19. Please discuss and reference, if this method has been used before.**

We did no use the WHO criteria for multiple reasons: first of all, patients with mild disease are not admitted to the hospital in the Netherlands, leaving us with only two categories. Secondly, the difference between moderate and severe disease according to the WHO guidelines is made based on respiratory rate (with a threshold of 30/min) and oxygen saturation (with a threshold of 90% on room air). Due to the nature of our data, which includes continuous respiratory rate values, we have observed that respiratory rate changes often and quickly. A patient could change between the moderate and severe group multiple times within one hour. Regarding the threshold for oxygen saturation, this threshold was made to use without room air. The vast majority of patients in our cohort however receive supplemental oxygen, so it would be almost impossible to decide in which category they belong. We could have used the situation at admission, however this does not reflect the aim of our study: to determine the change in circadian pattern during deterioration. The more conventional method to define deterioration would be intensive care admission. However, we do not believe that the time of admission to the intensive care adequately reflects the moment a patient becomes respiratory insufficient. A patient could have received 100% supplemental oxygen for hours without improvement of the situation, which unfortunately happened often during the covid-19 crisis. Furthermore, many patients had treatment restrictions, and 15L/min O2 was the highest level of care they were willing to receive or which was medically responsible.

Since we had such a specific aim and population, we decided to define a new endpoint. We hope this explanation clarifies our choice.

**6. Methods: “For quantitative assessment we divided the data in daytime (06:00-00:00) and nighttime (00:00-06:00).” The daytime period is proportionally much longer than the nighttime. Please discuss and reference, why this method was chosen and if this method has been published before.**

Thank you for this observation. Due to the new methodology, we have no longer defined daytime and nighttime.

**7. Methods: Data collection included the Charlson Comorbidity Index, but the group differences were not further discussed within the manuscript. The use of further disease severity scores for ICU patients (SOFA, GCS) would complement the author’s analysis.**

Thank you for this suggestion. Since our population is not an ICU population, but patients at the general ward, we have no routinely collected data on SOFA or GCS. Unfortunately, we also have not collected any other form of disease severity score. Instead we have tried to stratify our analysis based on disease severity: a cohort of patients who recovered without respiratory insufficiency, a cohort of patients who developed respiratory insufficiency, and a cohort of patients who died.

**8. Statistics: Although the authors nicely removed several abrupt deviations before analysis, the use of PNex measurement might not be picking up circadian trends very well. It is correlated, however results are very noisy. For a better understanding the analysis needs to be discussed and compared with other methods applied in previous publications examining vital signs. Why did the authors not perform a regular rhythmicity analysis and sine curve fit to better estimate the amplitude?**

Thank you very much for this helpful suggestion. Based on this comment and comments by the other reviewers, we have chosen to change our methodology altogether. We have fitted both a population mean cosinor model and a mixed-effect cosinor model to answer our different research questions. We hope our new methodology leads to better understanding.

**9. Discussion: Studying a clinical cohort of Covid-19 patients exclusively, the authors conclude that a general knowledge of circadian patterns might improve general monitoring- and alarm strategies. This affirmation is not supported by data including other disease entities and therefore needs to be discussed including more recent literature and trials examining circadian patterns in clinical cohorts.**

Daou M, Telias I, Younes M, Brochard L, Wilcox ME. Abnormal Sleep, Circadian Rhythm Disruption, and Delirium in the ICU: Are They Related? Front Neurol. 2020 Sep 18;11:549908. doi: 10.3389/fneur.2020.549908. PMID: 33071941
Lachmann G, Ananthasubramaniam B, Wünsch VA, Scherfig LM, von Haefen C, Knaak C, Edel A, Ehlen L, Koller B, Goldmann A, Herzel H, Kramer A, Spies C. Circadian rhythms in septic shock patients. Annals of Intensive Care. 2021 11: 64. PMID 33900485
Maas MB, Lizza BD, Abbott SM, Liotta EM, Gendy M, Eed J, Naidech AM, Reid KJ, Zee PC. Factors Disrupting Melatonin Secretion Rhythms During Critical Illness. Crit Care Med. 2020 Jun;48(6):854-861. PMID: 32317599
Maas MB, Iwanaszko M, Lizza BD, Reid KJ, Braun RI, Zee PC. Circadian Gene Expression Rhythms During Critical Illness. Crit Care Med. 2020 Dec;48(12):e1294-e1299. PMID: 33031153

Thank you very much for the suggested literature. We have used several to clarify and improve the discussion of our article. However, the suggested articles are all studies on critically ill patients in the intensive care unit. We feel strongly that the vital signs of critically ill patients at an intensive care unit cannot be compared to the vital signs of moderately ill patients at a low care ward, and that monitoring strategies for both wards are different, so we have avoided direct comparison. We did add several references of studies on general wards where wearables are used for continuous monitoring of covid-19 patients. Furthermore, we have highlighted why awareness of circadian patterns might aid interpretation of vital sign trends in clinical practice. The cited paper by van Rossum et. al. ((van Rossum et al., 2021) reference [24]), who found that the alarm strategy at a surgical ward could be improved by using different threshold for days and nights, underlines the potential of knowledge of circadian patterns to improve alarm strategies.

**Reviewer #2**

**The main issue is that the current study design does not support an analysis of risk for hypoxemic respiratory insufficiency as the authors propose. Evaluating different time periods a single cohort of patients, comparing a pre-hypoxemic “control” period and post-hypoxemic “outcome” period in those who developed hypoxemic respiratory insufficiency (defined in this study as >15L O2 therapy), we are able to establish only that these measurements differ in these two periods of time within these patients. To evaluate for risk of hypoxemic respiratory insufficiency, the authors must include a study population that did not develop hypoxemic respiratory insufficiency. This could be done in a variety of ways to create either a cohort or a case-control study design. The analysis should be revised to include such a cohort, or the manuscript needs to be rewritten with this significant change in mind. It would also be helpful to include references using the methods employed (daytime peak vs. nighttime nadir of physiologic data) to evaluate circadian patterns in the data.**

Thank you for pointing out this issue of the study design. Based on this comment, and comments made by the other reviewers, we have decided to apply a new analysis. We have included 3 cohorts: patients who recovered without experiencing respiratory insufficiency, patients who developed respiratory insufficiency and patients who died. We have not gone as far as to make a predictive model. We feel like this study is an explorative precursor study to see is a predictive model based on circadian pattern amplitude would be feasible. To better reflect this, we have adjusted the statement of objectives in the introduction.

Since we have abandoned the method of daytime peak and nighttime nadir, we have not included these references in the article.

**Abstract
- Please define “delta day” as a measurement in the abstract.**

**- Please revise to more accurately describe the logistic regression results (that VS changes are associated with worsened hypoxemia within in this select cohort of patients; they do not demonstrate risk of developing hypoxemia – as above, this cannot be evaluated unless the study also includes patients who did not develop hypoxemic respiratory insufficiency.)**

Since we have abandoned this method, the mentioned sentences are no longer part of the abstract.

**Methods**
**- p5. Please clarify - if sT measurements were not directly available then how were they studied?**

Skin temperature values were not available for clinical use. They were however stored for research purposes.

**- Please include how many COVID19 patients were excluded for lack of sensor data.**

We have added a flowchart of the patient inclusion (figure 1). We hope this will clarify the number of patients excluded during each stage.

**- p.6. Please correct “improbably data.”**

We have corrected improbably to improbable.

**- The authors excluded patients with < 48 hours of continuous data during a three-day period, except for the post-hypoxemic respiratory failure period. This suggests that patients who developed hypoxemic respiratory failure very quickly (eg after 1-2 days) or who died shortly after admission without developing hypoxemic respiratory failure, etc. would have been excluded. This biases the study population to those patients who had a relatively slow progression of disease and may bias the study population away from those admitted to the ICU (it is unclear whether remote monitoring continued after ICU admission?). The authors should consider revising the analysis, using a shorter time window for each “period”.**

Thank you for this helpful comment. We have redefined our cohorts, and have limited the amount of needed data to 4-hours. We believe this reduces the introduced bias. To be able to look at changes over time, we did include a three-day period of data.

**- Consider including a flow diagram to describe the total number of potentially eligible patients, and those excluded at each stage (e.g. as suggested by the STROBE statement).**

Thank you very much for this suggestion, we have included a flow chart (figure 1).

**- Including patient data in more than one time-period “cohort” and then comparing differences between time periods is problematic from a study design perspective. This introduces bias towards the null – which makes it more difficult to identify true differences in vital signs between cohorts. The authors should consider revising their compared time periods so that no overlap occurs.**

Thank you for this observation. As mentioned before, we have redefined the cohorts, limiting the introduced bias.

**- How accurately was the onset of hypoxemic respiratory insufficiency defined? It would be helpful to describe how this information was obtained, e.g. if this was time stamped (hour and minute) to ensure that the vital sign data was assigned correctly as pre- vs. post-intervention.**

The onset of respiratory insufficiency was defined as the first recording in the electronic patient record of either 15L/min oxygen therapy, high flow oxygen therapy, ventilation, or a cardiac arrest call. We have added this information to the data selection paragraph of the methods.

**- p.7. Were peaks and nadirs drawn from raw data? Typically, circadian analyses using peak/nadir are drawn from a cosinor or sinusoidal analysis that accounts for the entire dataset shifting + and – in a circadian fashion, which is less sensitive to random noise (e.g., Shoben Am J Epi 2011 for an excellent example.) If no cosinor analysis is applied, thenit seems like this peak-nadir measurement (PNex) will only provide the range of daytime vs. nighttime data. Please include references for using this measurement in circadian analyses.**

Thank you very much fort his comment. It has led us to revise our methodology, and we have now fitted a cosinor analysis (including references).

**Results:**

Thank you very much for the following comments regarding the results section. Since we have changed the methodology and therefore the results, these comments are no longer applicable. We have however tried to apply the feedback in general to the new result section.

**- p. 7. Please rename the 5 time periods; they are not truly “cohorts” as they include the same patients, and the patient data is actually duplicated across some of these analytic groups. Suggest using “time periods” or something similar.**

**- How do more patients have respiratory insufficiency than deterioration? This might be clarified by using more distinct/separated definitions of time periods for comparison and/or a flow diagram as suggested above.**

**- Table 3 sugests that day 1 vs. 2 of respiratory insufficiency and day 1 vs 3 of respiratory insufficiency were compared in logisitc regression. This is different from the analyses described in the Methods. Please clarify.**

**-p.9; was mean PNex compared quantitatively between stages using a statistical test? If so, consider adding these comparisons to table 2.**

**-p.9, lines 189-191 and 191-192 should be edited for accuracy. Since patients without hypoxemic respiratory insufficiency were not included in this analysis, we cannot evaluate whether this development was associated with the development of hypoxemia. All we know is that the delta PNex was associated with skin temperature in this select cohort of patients who will go on to develop respiratory insufficiency.**

**-Table 3: Table 3 compares the difference in Pnex between days 1 and 2 and days 1 and 3. Please clarify – is this limited to stage III patients? If so, please add this detail and the number of patients included to the title.**

**Discussion:
- I agree that Figure 1 suggests qualitatively that there is circadian variability given the cyclical nature of overall VS pattern. However, it’s hard to claim definitively that there is no circadian variability based on a subjective assessment (eg for the patients with mortality.) From figure 1, stage I and stage III do not necessarily look all that different – it is not clear what the authors used as a cut off for circadian rhythm presence vs. absence. If a quantitative analysis is possible, one should be included. If not, the discussion should reflect this uncertainty throughout.**

Thank you for this suggestion. We have added a quantitative analysis of rhythmicity to the methods of our study.

**- The Discussion should be edited in several places to accurately reflect the results presented in Table 3. This analysis supports only an association between PNex and skin temperature, not an association with hypoxemic respiratory failure.**

Since we have changed this analysis, these results are no longer part of the study.

**- If the PNex is not a validated method of measuring circadian amplitude, then the related portions of the discussion should be revised accordingly.**

Although other studies did use PNex to model circadian rhythms ((Davidson et al., 2021) reference [21]), we have decided not to use PNex as method but used a cosinor model instead. We have added to the discussion that the different methods might have different results.

**Figure 1
- Please provide a legend for the green, red, and black tracings. The caption says that the Y axis is hours; is this correct? (It seems like the X axis is hours?)**

Indeed, the X-axis was supposed to be hours, thank you for pointing this out. We have added the legend to the description of the figure.

**Figure 2
- Consider defining in the methods that you plan to compare day 1, 2 and 3 within the 5 time windows. Were any quantitative analyses applied to these 1/2/3 day comparisons? Clarifying why it was included and what these results mean would be helpful.**

Since we have changed this analysis, these comparisons are no longer part of the study.

Reviewer #3

*Major Comments*
**1. We ask the authors to explain in more detail the rational for the definition of each predefined stages/cohorts? Why did the authors did not perform a longitudinal statistical analysis? It would be interesting to know if circadian patterns change over time depending on specific covariates (covariate analysis: e.g. respiratory insufficiency as a covariate). We suggest a separate statistical methods paragraph. Why are p-values not provided within the manuscript. A review by an independent statistician may be helpful.**

Thank you for this valuable comment. We redefined the cohorts, and have paid attention to explaining why the new cohorts were chosen. Using the mixed-effect cosinor model, we have aimed to gain insight into changes of amplitude over time depending on the cohort (figure 3). Although this analysis does not provide prognostic information, we believe it does explore differences in circadian patterns over the days that might be used as input for prognostic studies.

**2. The applied definition of _a circadian pattern_ should be explained in the manuscript. Sentences like _A circadian pattern was observed in HR and RR during stages I-IV._ are unclear. When should clinicians deem a pattern as a _normal circadian rhythm_? Did the authors look for indicators of circadian disturbances when analysing the data? A longitudinal analysis of patterns comparing patients with and without developing respiratory insufficiency would be very interesting for the readership.**

Thank you for this feedback. We have chosen to change the method altogether, to avoid confusing and unclarity. We have added a quantitative measure of rhythmicity by doing a ‘goodness of fit’ test of the cosinor model. We have aimed to show longitudinal changes by using the mixed-effect cosinor model.

**3. The results section is very hard to "digest"; especially for readers who are not experts in the s very new field of research. We strongly suggest to amend the results section with figures, which illustrate the main results more clearly**.

Thank you very much for this suggestion. Since the methods have changed, the results section has changed too. We have added figure 3 and supplemental figure 1 to illustrate results.

**4. The authors conclude that circadian patterns of heart rate, respiratory rate and skin temperature could improve monitoring- and alarm strategies. At this stage of the manuscript, we do not think that this conclusion is supported by the data. We agree, that including informations of circadian profiles within in prediction models and monitoring algorithms may be an interesting and helpful approach in the future. We ask the authors to provide 1 or 2 concrete examples or clinical scenarios how this could be helpful in clinical decision making. However, the authors should be clear, that this is not supported by their data and should remove statements from the conclusion section.**

We agree that the improvement of monitoring- and alarm strategies was not the scope of this article and have removed this statement from the conclusion. In the paragraph ‘use in predictive modelling and clinical practice’ you will find a concrete example of how awareness of circadian patterns might influence clinical decision making.

**5. Page 10, Line 204: „The mean circadian pattern amplitude showed differences between stages, but only an increasing PNex of skin temperature was associated with developing respiratory insufficiency and with only a small effect size." Please provide a P-value. Was the association statistical significant?**

We have changed the analysis and provided p-values in the new result section.

***Further Comments*
- Page 3, Line 42-44: „Since September 2020, patients with covid-19 are treated with dexamethasone, which has a suppressive effect on the circadian pattern of the human metabolism." What does _suppressive effect on circadian pattern_ mean? Please explain.**

We agree with you and reviewer 1 that this statement is unclear and incorrect. We have changed the sentence to ‘..dexamethasone, which can affect the circadian pattern of the human metabolism depending on time of administration’ to better reflect that dexamethasone can either suppress or amplify circadian patterns, depending on the parameter and the timing of administration.

**- Page, Line 226: „The decrease of circadian rhythm might be due to severe illness and extreme physical stress, but could also have been influenced by age, comorbidities and medication. Furthermore, circadian rhythms are highly influenced by light input. As part of palliative care, patients were often relocated to single rooms with closed blinds for comfort. This might have played a role in the decrease of circadian pattern seen in all vital parameters.“ Please explain in more detail why circadian rhythm disturbances can occur in the context of severe diseases, e. g. closed eyes, reduced physical activity, inflammation etc.**

Thank you for this suggestion. We have clarified these statements with details and added several references.

**References**

Davidson, S., Villarroel, M., Harford, M., Finnegan, E., Jorge, J., Young, D., Watkinson, P., & Tarassenko, L. (2021). Day-to-day progression of vital-sign circadian rhythms in the intensive care unit. *Critical Care*. https://doi.org/10.1186/s13054-021-03574-w

Selvaraj, N., Nallathambi, G., Moghadam, R., & Aga, A. (2018). Fully Disposable Wireless Patch Sensor for Continuous Remote Patient Monitoring. *Proceedings of the Annual International Conference of the IEEE Engineering in Medicine and Biology Society, EMBS*. https://doi.org/10.1109/EMBC.2018.8512569

van Rossum, M. C., Vlaskamp, L. B., Posthuma, L. M., Visscher, M. J., Breteler, M. J. M., Hermens, H. J., Kalkman, C. J., & Preckel, B. (2021). Adaptive threshold-based alarm strategies for continuous vital signs monitoring. *Journal of Clinical Monitoring and Computing*. https://doi.org/10.1007/s10877-021-00666-4
