# Supplementary material for: Circadian patterns of heart rate, respiratory rate and skin temperature in hospitalized COVID-19 patients
Source: PLoS One. 2022 Jul 7;17(7):e0268065. doi: 10.1371/journal.pone.0268065 (PMC9262173; doi:10.1371/journal.pone.0268065)
Supplement: Attachment — Submitted filename: Rebuttal - Ciradian patterns in COVID-19.docx [file pone.0268065.s006.docx]

**Circadian patterns of heart rate, respiratory rate and skin temperature in hospitalized COVID-19 patients** - Response to reviewers’ and editors’ comments, second resubmission

Dear prof. dr. Oster, dear reviewers,

Thank you very much for the acceptance of our adjusted analysis. We are glad to see that you feel our manuscript has been improved because of it, and is now clear and easier to interpret. We would like to thank you for the additional suggestions for further improvement. We have addressed all questions and suggestions below.

We hope you deem our work now up to your standards, and that you will consider our manuscript for publication. If any further improvements need to be made, we will, of course, be happy to do so.

Looking forward to hearing from you,

Sincerely,

Harriët van Goor

h.m.r.vangoor-3@umcutrecht.nl

**Response to reviewers**

The authors have substantially revised their manuscript reflecting a complete revision of their analysis, now comparing circadian patterns in vital signs between three outcome-based groups: patients who recovered without respiratory insufficiency, those who developed respiratory insufficiency, and those who died. This analysis is much clearer and more readily interpreted. I have one remaining question regarding time frame definitions for analysis within each cohort, as well as some minor suggestions/clarifications.

**Introduction
1. P.4 – This paragraph could likely be shortened without losing substantive information. For example, “Assuming a physiological difference in vital sign values between night and day might be closer to reality than assuming equal values throughout the entire 24-hour cycle” may no longer fit the analysis employed in this manuscript.**

Thank you for the suggestion, we have critically reviewed the paragraph and have removed several sentences.

**2. p.4 – minor detail, consider adding the 3rd cohort (patients who did not develop respiratory insufficiency) to the description of your 2nd research aim.**

We have added the third cohort to the description of the research aim.

**Methods
1. p.6 – Please clarify the sentence beginning “Patients were included if…”. The “respiratory insufficiency resp. death” is confusing.**

We have clarified and elaborated on the choice of data for patients in each cohort.

**2. Suggest moving the “4 hours of data” requirement to earlier in the paragraph – you restricted this analysis to patients who had at least 4 hours of (continuous?) data within the time frame of interest.**

We decided to write down the methods in the same order as we analysed the data, to make reproduction of the study as easy as possible. Since we first selected the 72 hour timeframes, and later excluded those patients who appeared to have less than 4 hours of data within this timeframe, this is the chronological order of analysis.

**3. Because you analyzed serial days within the 72h window per patient, did you require 4 hours of data per day?**

No, we required 4 hours of data in total to include a patient in the population mean cosinor and rhythmicity test. For the stratification per day, a patient was only included in the day he/she had data during that day.

**4. How were you able to do the cosinor modeling for patients with only 4h of data per day? It seems like this would require more data.**

The cosinor model does not need a full cycle to estimate a cosinor curve. 4 hours of data, plus the indication where on the 24 hour cycle this data is located (the time) was enough to estimate the rest of the curve.

**5. Choosing comparable time frames in these three outcome-defined cohorts is challenging. The authors have chosen three different time frames during the admission, in which the patient physiology could be expected to be very different – 72h after admission for patients who recovered, 72h before respiratory failure in those patients, and 72h before death in those patients. Because LOS in the different cohorts was quite different, to help understand the “comparability” of these patients, providing some information about where this window fell in the average length of stay for each cohort would be helpful. (For example – the 72h is the first 3 days out of an average 6 day LOS for the recovery cohort. But was it also on average the first 3 days for the respiratory failure cohort – e.g., when in the hospital stay did the respiratory failure occur?) This matters because patients who have been hospitalized longer have more opportunity for circadian disruption, and you may be finding changes in this cohort that are attributable solely to longer LOS/later time of evaluation and not attributable to clinical decline. If this is the case, this source of bias would need to be explicitly discussed in the discussion (and could be addressed in multiple locations in the discussion, as it may help explain the inconsistent findings between the respiratory failure and mortality cohorts, as well as the presence of respiratory and temperature variability only in the recovery cohort.) One could also consider trying to address this analytically, e.g. by matching recovery patients to patients in the other cohorts based on LOS, but this is probably not worth doing.**

Thank you for your thorough consideration of the implications of timeframe selection. We share your opinion that the comparability of patients would be less if the timeframes were selected from different parts of the admission. Because respiratory insufficiency usually occurs within the first 72 hours of admission (median 33 hours), we chose the first 3 days of admission for the recovery (“control”) group too. We realise we failed to mention this in earlier versions of the manuscript, but we have now added it to the methods on page 6/7 of the manuscript.

Regarding the mortality cohort, mortality usually occurred later than 72 hours after admission. The comparability of this cohort with the other two cohorts could indeed be questioned. We have added this to the discussion on page 13 and to the limitations of the study on page 14, including the consideration to use a case-control design matched on length of hospitalization in future research.

**Results
1. p.10 - Please clarify “stratified by day”. Do you mean stratified by cohort (as in the title of figure 3)?**

Actually, figure 3 presents the cosinor characteristics stratified by cohort and by day. We have clarified this both in the manuscript and in the title of figure 3.

**Minor comments
- Some editing for grammar would be helpful, e.g. p. 3 “circadian rhythm become increasingly more pronounced”, p.4 “three…research questions”, etc.**

Thank you for your acuity. We have checked the entire manuscript and have corrected several grammatical mistakes.
